# Cardiomyocyte-specific deletion of TLR4 attenuates angiotensin II-induced hypertension and cardiac remodeling

**DOI:** 10.3389/fcvm.2023.1074700

**Published:** 2023-03-24

**Authors:** Drew Theobald, Anand R. Nair, Srinivas Sriramula, Joseph Francis

**Affiliations:** ^1^ Department of Pharmacology and Toxicology, Brody School of Medicine at East Carolina University, Greenville, NC, United States; ^2^ Comparative Biomedical Sciences, School of Veterinary Medicine, Louisiana State University, Baton Rouge, LA, United States

**Keywords:** hypertension, TLR4, cardiac hypertrophy, inflammation, angiotensin II

## Abstract

Toll-like receptor 4 (TLR4) is an integral factor in the initiation of the innate immune response and plays an important role in cardiovascular diseases such as hypertension and myocardial infarction. Previous studies from our lab demonstrated that central TLR4 blockade reduced cardiac TLR4 expression, attenuated hypertension, and improved cardiac function. However, the contribution of cardiac specific TLR4 to the development of hypertension and cardiac remodeling is unknown. Therefore, we hypothesized that cardiomyocyte specific knockdown of TLR4 would have beneficial effects on hypertension, cardiac hypertrophy, and remodeling. To test this hypothesis, cardiomyocyte-specific TLR4 knockdown (cTLR4KO) mice were generated by crossing floxed TLR4 mice with Myh6-Cre mice, and subjected to angiotensin II (Ang II, 1 µg/kg/min or vehicle for 14 days) hypertension model. Blood pressure measurements using radio telemetry revealed no differences in baseline mean arterial pressure between control littermates and cTLR4KO mice (103 ± 2 vs. 105 ± 3 mmHg, *p* > 0.05). Ang II-induced hypertension (132 ± 2 vs. 151 ± 3 mmHg, *p* < 0.01) was attenuated and cardiac hypertrophy (heart/body weight; 4.7 vs. 5.8 mg/g, *p* < 0.01) was prevented in cTLR4KO mice when compared with control mice. In addition, the level of myocardial fibrosis was significantly reduced, and the cardiac function was improved in cTLR4KO mice infused with Ang II. Furthermore, cardiac inflammation, as evidenced by elevated gene expression of TNF, IL-6, and MCP-1 in the left ventricle, was attenuated in cTLR4KO mice infused with Ang II. Together, this data revealed a protective role for cardiomyocyte-specific deletion of TLR4 against Ang II-induced hypertension and cardiac dysfunction through inhibition of proinflammatory cytokines.

## Introduction

Hypertension is the foremost risk factor for the pathogenesis of cardiovascular disease (11). The prevalence of uncontrolled hypertension is exceedingly high and is continuing to rise, even with the use of readily available antihypertensive therapeutics and lifestyle modifications. Failure to control blood pressure to recommended levels can result in an augmented cardiovascular load which can cause chronic inflammation and oxidative stress, resulting in maladaptive cardiac remodeling and fibrosis (15). In hypertensive human patients, it has been shown that disproportionate cardiomyocyte hypertrophy and myocardial fibrosis results in increased myocardial stiffness and impaired systolic and diastolic function (18, 41). Given the impact of hypertension worldwide, there is an urgent need for novel antihypertensive therapeutic modalities.

Many studies have highlighted the importance of the renin-angiotensin system (RAS) in the development of hypertension and with specific focus on cardiovascular inflammation and fibrosis (7, 26). Additionally, there is overwhelming evidence that the use of angiotensin receptor blockers (ARB) and angiotensin converting enzyme inhibitors (ACEi) have the ability to reduce cardiovascular disease mortality and improve the overall quality of life for hypertensive patients by improving cardiac function and averting further cardiac remodeling (42). Recent studies have shown that hypertensive patients have elevated proinflammatory cytokine profiles, elevated reactive oxygen species production and vascular responses to lipopolysaccharide (LPS) (10, 31, 38). Interestingly, toll like receptor 4 (TLR4) is a major receptor that responds to LPS and has recently been shown to be elevated during hypertension in the brain and vasculature (2, 6, 7). Therefore, TLR4 may be a potentially unexplored antihypertensive therapeutic target.

The human immune system relies on pattern recognition receptors (PRRs), capable of recognizing different pathogen-associated molecular patterns (PAMPs) and damage-associated molecular patterns (DAMPs) (25). Of the known PRRs, Toll-like receptors (TLRs) are a family of receptors which recognize DAMPs and PAMPs and activate a downstream signaling cascade which eventually develops into an inflammatory response. Of the known 13 TLRs, TLR4 has been extensively studied within hypertension and has been shown to contribute to the progression of inflammatory responses in the etiology of the disease (8, 28, 34). We previously showed that direct intra-paraventricular nucleus infusion of VIPER, a TLR4 inhibitor, was able to decrease TLR4 expression in the PVN, as well as attenuate blood pressure and inflammatory response (5). In addition, inhibition of TLR4 in the PVN further decreased inflammatory cytokines and was responsible for diminished sympathoexcitation in SHRs, which led to a decrease in cardiac hypertrophy (6).

Studies have suggested that continuous activation of TLR4 induces the generation and liberation of proinflammatory cytokines, chemokines, and costimulatory factors which lead to low-grade chronic inflammation in the heart (9, 29). Further evidence has shown that TLR4 protein expression is increased in the hearts of individuals with diagnosed hypertension (3, 12). Within the heart specifically, Yang et al. showed that there is a hypertrophic response in cardiomyocytes of the heart, which is modulated by TLR4-mediated MyD88-dependent nuclear factor-kappa B (NF-κB) pathways (43). Cardiomyocyte hypertrophy is one of the initial responses by which the heart abates the stress on the LV wall imposed by pressure overload. The hypertrophic effect stimulates intracellular signaling cascades which exacerbate gene expression and protein synthesis. This consequently increases overall protein quantities in the magnitude and configuration of force-generating units (sarcomeres), resulting in an increase in the size of individual cardiomyocytes (22). Although it is known that TLR4 inhibition in the PVN is beneficial, TLR4 inhibition in the heart, and specifically within cardiomyocytes, has still yet to be fully studied. In this study we investigate whether cardiomyocyte specific TLR4 deletion will be able to attenuate the profibrotic, proinflammatory, deleterious cardiac function and physiological measurements of angiotensin (Ang) II-induced hypertension.

## Materials and methods

### Animals

Adult (10–12 weeks old, 25–30 g) male cardiomyocyte specific TLR4 knock-down mice (cTLR4KO) and their controls (WT) were used for experiments. For TLR4 deletion in the heart specifically in cardiomyocytes, (TLR4^flox/flox^) [B6(Cg)-Tlr4^tm1.1^Karp/J procured from Jackson laboratories] and was crossed with Myh6-cre-Cre^+/−^ strain. The cTLR4KO mice are fertile and devoid of gross anatomy defects. All animals were housed in a temperature- and humidity-controlled animal care facility with a 12-hour dark/light cycle. The mice were fed standard chow and water *ad libitum*. All animal procedures were approved by the Louisiana State University School of Veterinary Medicine Animal Care and Use Committee (#09–008) and East Carolina University Animal Care and Use Committee (AUP #W254) and are in agreement with the National Institutes of Health Guide for the Care and Use of Laboratory Animals.

### Mouse genotyping protocol

Genomic DNA was isolated from tail snips and genotyping of mice was performed using REDExtract-N-Amp Tissue PCR kit (Sigma). The TLR4 knockout allele was detected by primers (5′ to 3′) - TGATGGTGTGAGCAGGAGAG and TGACCACCCATATTGCCTATAC. Thermal cycler conditions used were 95°C for 5 min; followed by 40 cycles of 95°C for 15 s, 60°C for 30 s, 68°C for 45 s; 72°C for 5 min. Allele-specific PCR products were identified using 3% agarose gel electrophoresis.

### Experimental design

WT and cTLR4KO mice were implanted with radio-telemetry transmitters for blood pressure measurements as described previously (36, 37). Mice were anesthetized with isoflurane (2%) in an oxygen flow (1 L/min) and positioned on a heating pad to maintain body temperature around 37.5°C. Mice were implanted with telemetry transmitters (PA-C10, Data Sciences International) for conscious blood pressure recording. Pre- and post-operative care included a buprenorphine (0.05 mg/Kg, sc) injection to relieve pain. After a week of recovery from the surgery, baseline blood pressure was recorded for 3 days. Then, mice were implanted subcutaneously with osmotic minipumps (Alzet Model 1,002) to deliver either Ang II (1 µg/kg/min, Sigma Aldrich, A9525) or vehicle (0.9% saline) for 14 days. The mice groups are as follows: (1) WT + Saline (control mice infused with saline); (2) WT + Ang II (WT mice infused with chronic Ang II); (3) cTLR4KO + saline (knockout mice infused with saline); (4) cTLR4KO + Ang II (knockout mice infused with chronic Ang II). After 14 days, the mice were euthanized by decapitation under deep isoflurane anesthesia and the hearts were collected for further analyses.

### Echocardiography

At the end of 2-week Ang II-infusion period, the left ventricular morphology and function were evaluated noninvasively by transthoracic echocardiography. Mice were anesthetized with 1.5% isoflurane and short-axis M-mode echocardiograms on the left ventricle (LV) were performed using a Toshiba Aplio SSH770 (Toshiba Medical, Tustin, CA). The parameters fractional shortening (FS) and left ventricular anterior wall dimension during diastole (LVAWd) were all determined from the M-mode images.

### Gene expression analysis by quantitative real time RT-PCR

Total RNA was isolated from left ventricular tissue with Direct-zol RNA Miniprep Plus kit with on column DNA digestion (Zymo Research). Real Time RT-PCR amplification reactions were performed with Power SYBR Green RNA-to-C_T_ one-step Kit (Life Technologies) in triplicate using real time PCR machine (ABI Prism 7,900, Applied Biosystems). The specificity of SYBR green assays were confirmed by dissociation curve analysis. Data were normalized to GAPDH expression by the 2^−(ΔΔCT)^ comparative method and gene expression was presented as a fold change compared to control.

### Immunofluorescence staining

Anesthetized mice were perfused transcardially with PBS (0.1 M, pH 7.4) for 5 min followed by 4% paraformaldehyde in PBS (0.1M, pH 7.4) for 10 min as described previously (37). The hearts were collected and post fixed for at least 2 h in 4% paraformaldehyde in PBS (0.1 M, pH 7.4). The hearts were cryoembedded in OCT and then using a cryostat, 5 µm sections were cut and transferred onto pre-chilled slides. The slides were airdried for 10 min, followed by incubation with blocking buffer (5% donkey serum in PBS containing 0.2% Tween-20) for 1 h at room temperature. Then sections were incubated with primary antibodies (TLR4, 48–2,300 Invitrogen, 1:500 dilution) at 4°C overnight in a humidifying chamber. Immunolabeling was done with appropriate conjugated secondary antibody (Donkey anti-Rabbit Alexa Fluor Plus 594, A32754 Invitrogen, 1:1,000 dilution) for 60 min at room temperature. For determining cross-sectional area of cardiomyocytes, the cell membranes were labelled with wheat germ agglutinin (WGA) conjugated with Alexa Fluor 488 (#W11261, Invitrogen, 1 mg/ml, 1:200 dilution) for 10 min. Sections were mounted on glass slides with ProLong Diamond Anti-Fade Mount (Invitrogen). The images were captured using a fluorescence microscope (Keyence/Echo revolve). The cross-sectional area of cardiomyocytes was quantified by a blinded investigator using ImageJ software (NIH).

### Protein expression analysis by western blot

Western blots analyses for protein expression were performed on left ventricle homogenates, as described previously (37). Left ventricular tissue was homogenized with RIPA lysis buffer (Thermo Scientific) with protease and phosphatase inhibitors cocktail (Roche) and protein lysates were separated by centrifugation at 12,000 g and 4°C for 15 min. Protein concentration in tissue homogenates was determined using a Pierce BCA protein assay kit (Thermo Scientific). Equal amounts of protein (30 µg) were separated by SDS-PAGE on 4%–15% Mini-PROTEAN TGX gels (Bio-Rad) and transferred on to PVDF membrane and blocked with 1% BSA in TBS-T at room temperature for 60 min. The membranes were incubated overnight with primary antibody against TLR4 (48–2,300 Invitrogen, 1:1,000 dilution). Immunodetection was accomplished by incubation with IRDye secondary antibodies for 1 h at room temperature and imaged using Odyssey CLx imaging system (Licor). Protein expression of β-Actin or GAPDH was used as a loading control. The density of protein bands was quantitatively analyzed by Image J software (NIH) and expressed as a relative ratio against the loading control.

### Measurement of collagen

To determine collagen content as a measure of cardiac fibrosis, hearts were embedded, cut (5 µm thickness), and stained with Masson's Trichrome stain. The images were captured with a digital imaging system and quantified for collagen content by a blinded investigator using Image J software (NIH). A minimum of 5 images per heart were analyzed from each animal in the control and Ang II-infusion groups.

### Measurement of NF-κB activity

NF-κB activation was measured using a DNA binding assay as described previously (30, 37). Hearts were harvested from the mice and nuclear extracts from left ventricular tissue were collected using a nuclear extraction kit (Active Motif). Protein concentrations were then quantified using a Pierce BCA protein assay kit (Thermo Scientific). Equal amounts of nuclear extract proteins were utilized in a colorimetric NF-κB assay kit specific for the activated form of the p65 subunit of NF-κB (TransAm NF-κB p65, Active Motif) following the manufacturer's instructions.

### Statistical analysis

Data are presented as mean ± standard errors of the means. Experiments involving two groups were compared using unpaired, 2-tailed *t*-test. Multiple comparisons were made using two-way ANOVA followed by Bonferroni's *post hoc* analysis. Mean arterial pressure data was analyzed by two-way repeated measures ANOVA with Tukey's multiple comparisons test. Statistical analyses were performed using GraphPad Prism 7 (GraphPad Software). Values of *p* < 0.05 were considered statistically significant.

## Results

### TLR4 expression is increased in the heart in hypertension

**Figure 1:**
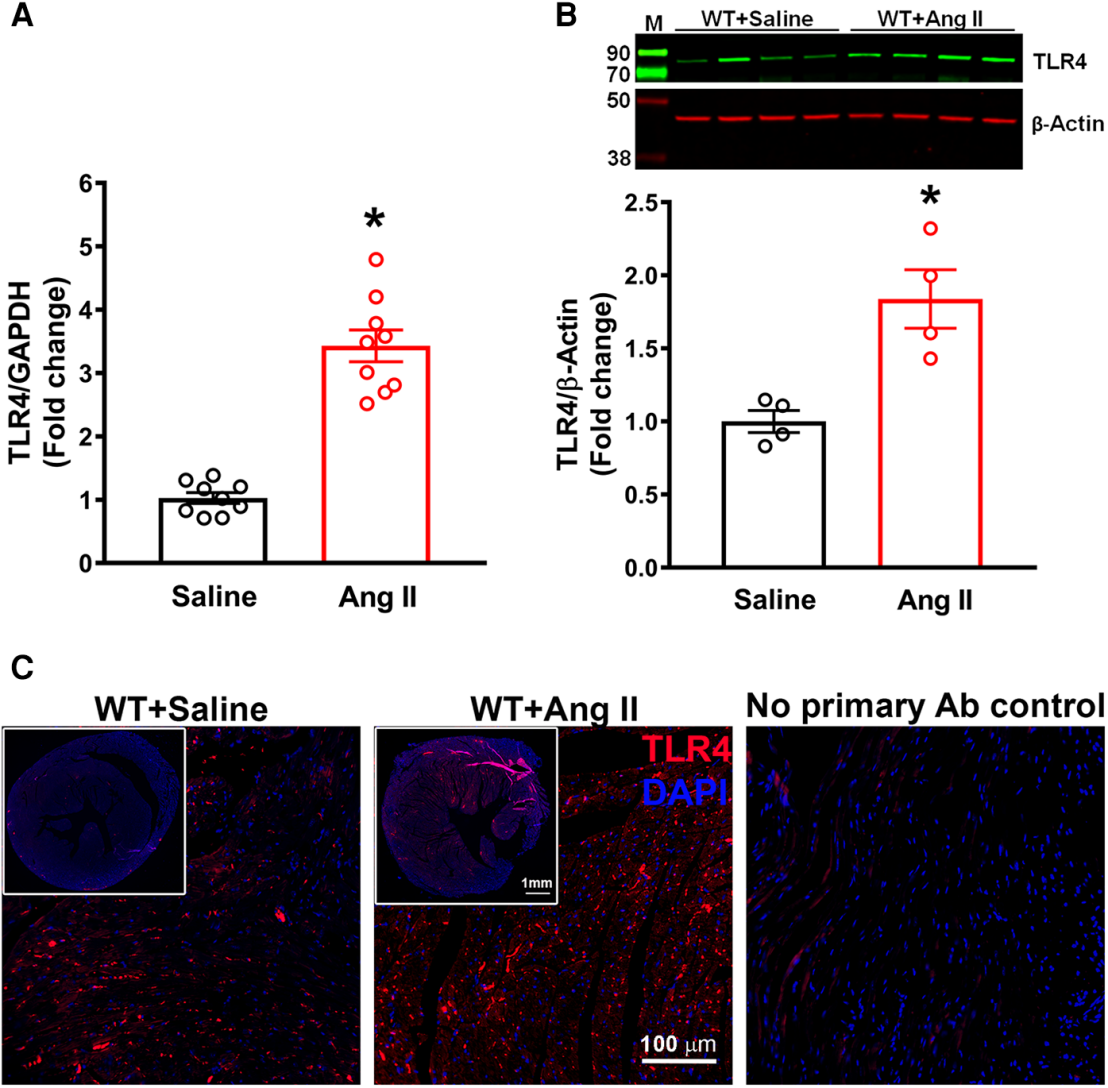
Cardiac TLR4 expression is increased in angiotensin II-induced hypertension. (**A**) Gene expression measured by real time RT-PCR shows a significant increase in cardiac TLR4 mRNA after 14 days of angiotensin (Ang) II treatment (*n* = 9/group, unpaired, 2-tailed *t*-test, *p < 0.05 vs. saline). (**B**) Representative images from western blot analysis and quantification reveals an increase in TLR4 protein expression in heart lysates from hypertensive mice (*n* = 4/group, unpaired, 2-tailed *t*-test, *p < 0.05 vs. saline controls). (**C**) Immunofluorescence staining reveals a largely elevated expression of TLR4 (red) in hypertensive mice compared with saline infused controls.

Initially, we determined the changes in expression of TLR4 in the hearts of hypertensive mice. TLR4 gene expression was significantly increased after 14 days of Ang II infusion when compared with WT mice (Figure 1A, *p* < 0.05). Moreover, TLR4 protein expression measured by western blot analysis was also significantly augmented in Ang-II treated mice when compared to WT mice (Figure 1B, *p* < 0.05). Immunofluorescence staining of heart sections with TLR4 specific antibody showed an elevation in expression within the heart in hypertensive mice compared to WT mice (Figure 1C). Corresponding negative control further revealed the specificity of the antibody.

### Cardiomyocyte-specific TLR4 deletion mice attenuates Ang II-induced hypertension

**Figure 2:**
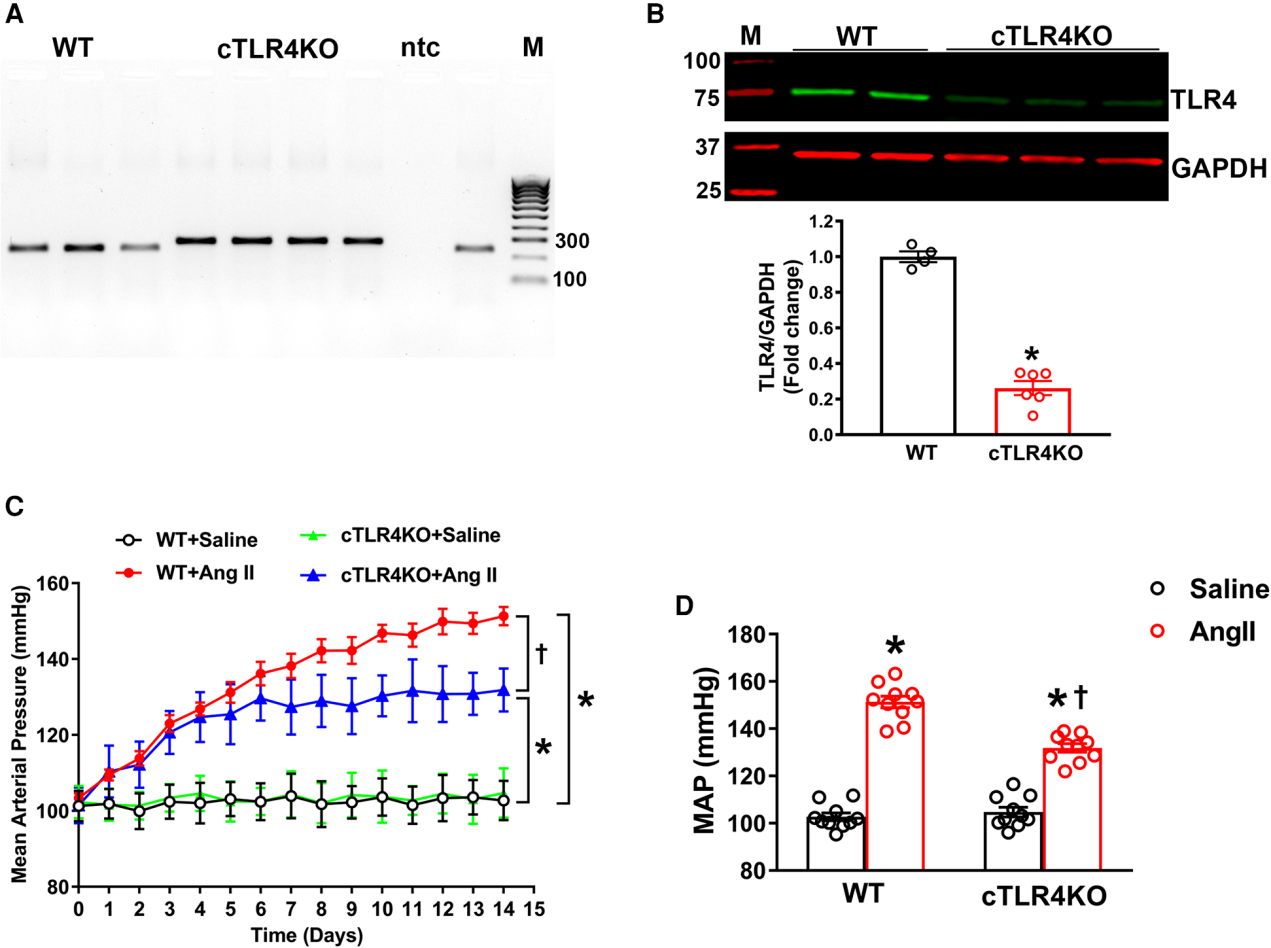
Cardiomyocyte-specific TLR4 deletion attenuates angiotensin II-induced hypertension. (**A**) Mouse genotyping PCR using genomic DNA extracted from tail snips confirms that cardiomyocyte-specific TLR4 deletion (cTLR4KO) mice contain only the cTLR4KO band (300 bp, samples 4–7), as wild-type (WT) mice do not exhibit this band (samples 1–3.9). ntc, no template control; M, molecular weight marker. (**B**) Representative western blot image and quantification data indicate a significant reduction in cardiac TLR4 expression in cTLR4KO mice compared to WT mice (*n* = 4–6/group, unpaired, 2-tailed *t*-test, *p < 0.05 vs. WT + Saline). (**C**) Time course of blood pressure measurement using radiotelemetry showing angiotensin II (Ang II) infusion significantly increased the mean arterial pressure (MAP) in WT mice, however this effect in cTLR4KO mice was significantly attenuated (*n* = 10, repeated measures two-way ANOVA, *p < 0.05 vs. WT + Saline, †*p* < 0.05 vs. WT + Ang II). (**D**) MAP at day 14 of Ang II-infusion (*n* = 10, two-way ANOVA, *p < 0.05 vs. WT + Saline, †*p* < 0.05 vs. WT + Ang II).

To explicitly ablate TLR4 in cardiomyocytes, homozygous TLR4^f/f^ mice were bred with Myh6-Cre transgenic mice expressing nuclear-localizing Cre recombinase under the control of the mouse Myh6 (myosin, heavy polypeptide 6, cardiac muscle, alpha; alpha-MHC) promoter, which enabled Cre recombinase to be expressed specifically in cardiomyocytes. The heterozygous TLR4^f/+^; Myh6-Cre mice were healthy and viable and were bred with TLR4^f/f^ mice to obtain homozygous TLR4 deletion mice (TLR4^f/f^; Myh6-Cre, hereafter referred to as cTLR4KO) (Figures 2A,B). The TLR4 deletion in the cTLR4KO mice was verified using Western blot analysis. To determine the effects of cardiomyocyte specific deletion of TLR4 on blood pressure in response to Ang II infusion we measured mean arterial pressure (MAP) evaluated by radiotelemetry. The baseline MAP was similar in WT and cTLR4KO mice as shown in Figure 2C, Ang II-infusion progressively increased MAP in the WT mice, and mice with cardiomyocyte specific deletion of TLR4 showed an attenuated response to the increase in MAP induced by Ang II. As shown in Figure 2D, the quantification of MAP data on day 14 shows that Ang II-infusion significantly raises the MAP in WT mice compared to saline infused controls, suggesting that TLR4 is in part required for the development of Ang II hypertension. This increase in MAP was attenuated within CTLR4KO mice that received Ang II-infusion.

### Cardiomyocyte-specific TLR4 deletion reduces Ang II-induced cardiac hypertrophy

**Figure 3:**
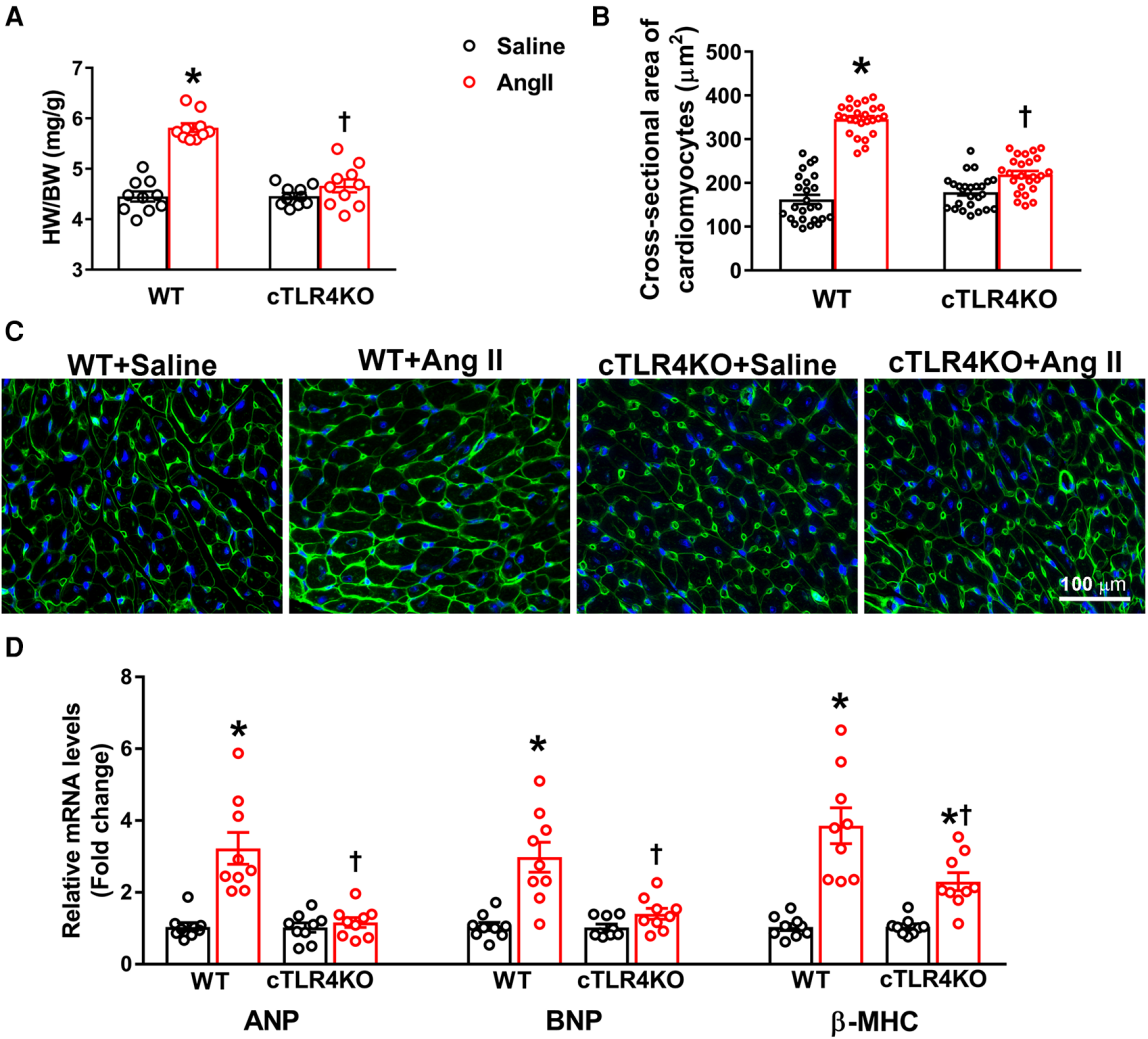
Cardiomyocyte-specific TLR4 deletion abrogates angiotensin II-induced cardiac hypertrophy. (**A**) Cardiac hypertrophy was measured by calculating heart weight/body weight (HW/BW) ratio. Ang II increased heart weight to body weight in WT mice, but this effect was blunted in cTLR4KO mice (*n* = 10, two-way ANOVA, *p < 0.05 vs. WT + Saline, †*p* < 0.05 vs. WT + Ang II). (**B**) To visualize cardiomyocyte hypertrophy, we used wheat germ agglutinin immunofluorescence staining to measure cardiomyocyte cross-sectional area (µM^2^). Quantification data showing a significant increase in cardiomyocyte cross-sectional area in WT mice treated with Ang II, which was attenuated in cTLR4KO mice (*n* = 4 mice/group, two-way ANOVA, *p < 0.05 vs. WT + Saline, †*p* < 0.05 vs. WT + Ang II). (**C**) Representative heart images showing immunofluorescence staining with wheat germ agglutinin. (**D**) Real time RT-PCR was used to measure gene expression of natriuretic peptides ANP, BNP and β-MHC. In hypertensive WT mice there is a significant elevation in these natriuretic peptides, which is attenuated in cTLR4KO mice (*n* = 9, two-way ANOVA, *p < 0.05 vs. WT + Saline, †*p* < 0.05 vs. WT + Ang II).

Cardiac hypertrophy is an adaptative mechanism and marker for cardiovascular disease in patients with hypertension. To evaluate cardiac hypertrophy, we collected the heart weight to body weight ratio in WT and cTLR4KO mice (Figure 3A). As expected, Ang II notably increased cardiac hypertrophy in WT mice but was attenuated in cTLR4KO mice as shown by heart weight to body weight ratios. To investigate the specific effects of cardiomyocytes in developing cardiac hypertrophy further, we measured the average cardiomyocyte cross sectional area (CSA) in heart sections that have been stained with wheat-germ agglutinin and quantified mRNA transcription of selected cardiac hypertrophy-related genes. We found that when compared to saline-infused WT mice, mice infused with Ang II showed a significant increase in CSA (16.20 ± 0.26 mm vs. 13.8060.34 mm, *p* < 0.05, Figures 3B,C), however, this increase was blunted in cTLR4KO mice with Ang II-infusion. This data suggests the direct role of TLR4 in cardiomyocytes and their effect on cardiac hypertrophy. In Ang II-infused WT hearts, gene expression analysis revealed a considerable increase in mRNA levels of hypertrophic markers ANP, BNP and β-MHC, further indicating a cardiac hypertrophic phenotype (Figure 3D). However, the mRNA expression levels of these markers were unchanged in cTLR4KO mice indicating protection from the Ang II-induced cardiac hypertrophy.

### Cardiomyocyte-specific TLR4 deletion reduces Ang II-induced cardiac fibrosis

**Figure 4:**
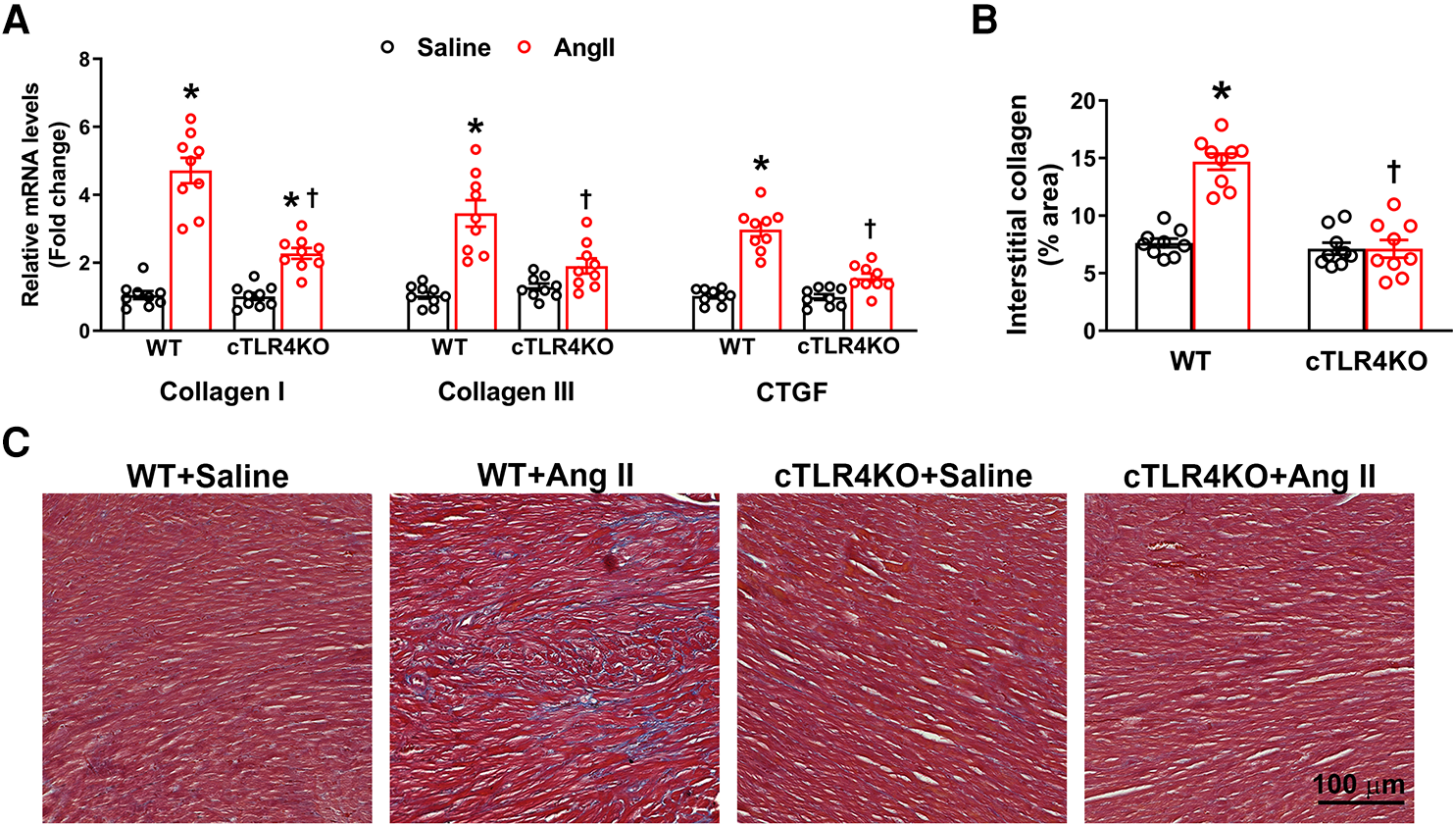
Cardiac-specific TLR4 deletion attenuates angiotensin II-induced cardiac fibrosis. (**A**) Gene expression of profibrotic markers (Collagen I, Collagen III, and CTGF) in the left ventricle tissue was measured using real time RT-PCR. Increased profibrotic gene expression was observed in hypertensive WT mice, but this increase was attenuated in cTLR4KO mice with Ang II-infusion. (**B**) Changes in collagen deposition leading cardiac fibrosis was analyzed by Masson's Trichrome staining. Mean interstitial collagen content in the left ventricular sections indicate an increased fibrosis in WT mice infused with Ang II. This increase in collagen deposition in the heart was attenuated in cTLR4KO mice receiving Ang II infusion. (*n* = 3 mice/group, Two-way ANOVA, *p < 0.05 vs. WT + Saline, †*p* < 0.05 vs. WT + Ang II). (**C**) Representative images of left ventricle sections stained with Masson's Trichrome staining indicating collagen fibers (blue in color).

Increases in cardiac fibrosis and profibrotic gene expression is a characteristic of Ang II-induced hypertension. Therefore, we measured markers of profibrotic gene expression, collagen I, collagen III, and connective tissue growth factor (CTGF, CCN2). As expected, Ang II increased gene expression of collagen I, collagen III and CTGF in WT mice, but this effect was attenuated in cTLR4KO mice (Figure 4A). To further investigate cardiac fibrosis, we measured interstitial collagen deposition using Masson's trichrome staining, where collagen fibers are stained blue. Ang II treated WT mice demonstrated a significant increase in collagen deposition (Figures 4B,C, *p* < 0.05), which was abrogated in cTLR4KO mice. This data suggests cardiomyocyte specific TLR4 deletion prevents Ang II-induced cardiac fibrosis.

### Cardiomyocyte-specific TLR4 deletion attenuates Ang II-induced cardiac dysfunction

**Figure 5:**
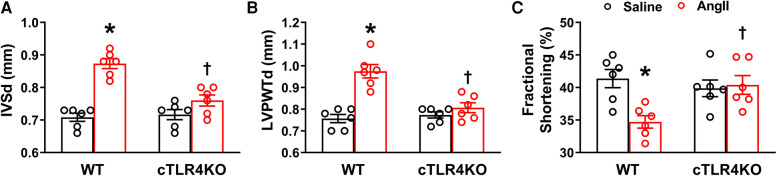
Cardiomyocyte-specific TLR4 deletion attenuates angiotensin II-induced cardiac dysfunction. Echocardiography was used to evaluate cardiac function following Ang II infusion in hypertensive mice. In WT mice, Ang II significantly increased (**A**) interventricular septal thickness end diastole (IVSd) and (**B**) left ventricular posterior wall thickness end diastole (LVPWTd), while decreasing (**C**) fractional shortening %. However, these changes were reversed in cTLR4KO mice with Ang II infusion. (*n* = 6 mice/group, two-way ANOVA, *p < 0.05 vs. WT + Saline, †*p* < 0.05 vs. WT + Ang II).

We used echocardiography to determine the consequences of cTLR4 deletion on left ventricular function in mice. In WT mice, Ang II significantly augmented interventricular septal end diastole (IVSd), left ventricular posterior wall end diastole (LVPWTd), and fractional shortening %, however this effect was attenuated in cTLR4KO mice (Figure 5). These results further indicate that cTLR4KO mice have a preserved cardiac function when compared with WT mice treated with Ang II.

### Cardiomyocyte-specific TLR4 deletion attenuates Ang II-induced inflammation in the heart

**Figure 6:**
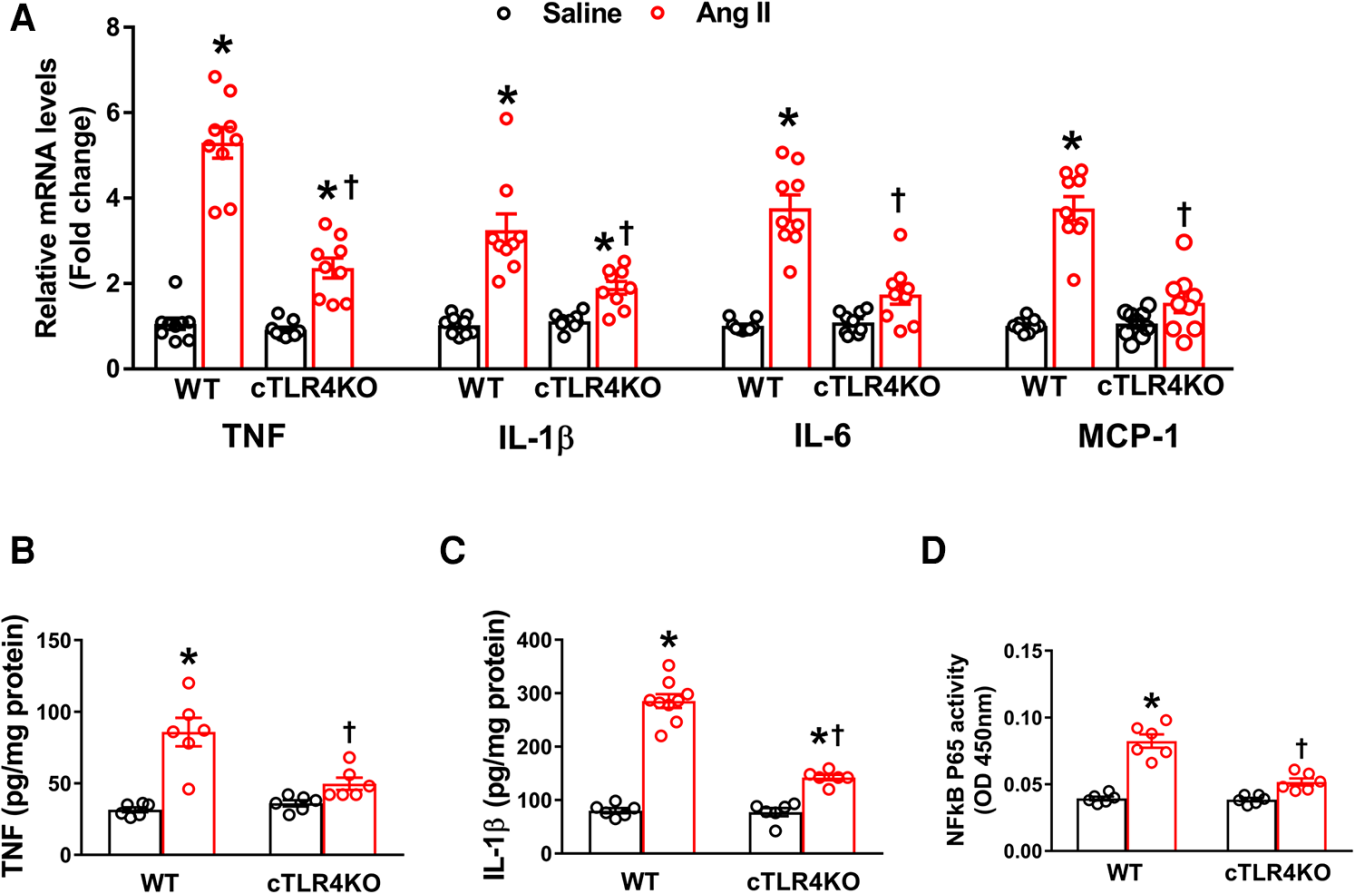
Cardiomyocyte-specific TLR4 knockdown reduces mRNA expression of pro-inflammatory markers. (**A**) Angiotensin II (Ang II) infusion for 2 weeks significantly increased TNF, IL-1β, IL-6, and MCP-1 mRNA expression in the left ventricle cardiac tissue indicating an increased inflammatory state. This effect was attenuated in cTLR4KO mice (*n* = 9, two-way ANOVA, *p < 0.05 vs. WT + Saline, †*p* < 0.05 vs. WT + Ang II). ELISA analysis of left ventricular lysates showed that Ang II increased (**B**) TNF and (**C**) IL-1β in WT mice but these increases were blunted in cTLR4KO mice (*n* = 6–9, two-way ANOVA, *p < 0.05 vs. WT + Saline, †*p* < 0.05 vs. WT + Ang II). (**D**) NF-κB binding activity measured in the left ventricle nuclear extracts exhibited a significantly increased cardiac NF-κB p65 activity in WT mice but not in cTLR4KO mice with Ang II-infusion (*n* = 6, two-way ANOVA, *p < 0.05 vs. WT + Saline, †*p* < 0.05 vs. WT + Ang II).

To determine the effect of Ang II on the production of cytokines and chemokines in the heart, we used real time RT-PCR to measure the mRNA expression of TNF, IL-6, IL-1β, and the chemokine MCP-1 in the left ventricle. Mice infused with Ang II demonstrated an increase in pro-inflammatory cytokines' TNF, IL-6 and IL-1β, and the chemokine MCP-1 gene expression within the left ventricle of WT mice but was prevented in cTLR4KO mice (Figure 6A). Serum ELISA showed an increase in TNF and IL-1B in WT mice treated in Ang II, but cTLR4KO mice were protected from this effect (Figure 6B, C). As a result of increased proinflammatory cytokine production, transcription factors such as NF-κB can become activated. We measured NFκB levels using a specific NF-κB activity assay. Our results showed that Ang II increases NF-κB activity in WT mice but is attenuated in cTLR4KO mice (Figure 6D). These findings further suggest that cardiomyocyte specific TLR4 deletion reduces inflammation within the left ventricle in Ang II-induced hypertension.

## Discussion

The inability of hypertensive patients to adequately maintain their blood pressure levels with available therapeutics indicates an urgent need for novel targets for the treatment of hypertension and ultimately cardiovascular disease. Myocardial fibrosis is a crucial pathological process in the development of cardiac hypertrophy and fibrosis and can be a direct result of chronic hypertension (20). These physiological changes result in the progression of cardiovascular disease in hypertensive patients (20). TLR4 has been identified as being directly involved in the upregulation of inflammation and fibrosis (4). Prior studies have shown the beneficial effects of TLR4 inhibition, specifically within the brain (16, 24). To our knowledge, our study is the first to show that cardiomyocyte specific TLR4 deletion attenuates the effects of angiotensin II on cardiac hypertrophy, fibrosis, function, and inflammation.

Previously, our laboratory showed that centrally blocking TLR4 improved cardiac function as well as attenuated myocardial inflammation in genetic and angiotensin II-induced hypertension animal models (5, 6). In this study, we build upon these findings and focus specifically on TLR4 inhibition in cardiomyocytes, the major contractile cells of the heart in Ang II-induced hypertension. TLR4 has been previously shown by our laboratory and others to be expressed in the heart (1, 12, 17, 40). To confirm these findings in Ang II-induced hypertension, we measured TLR4 gene and protein expression in the heart and showed a significant elevation in TLR4. Furthermore, we visualized the expression of TLR4 in the heart using immunofluorescence staining and could identify an increase in protein expression. Therefore, our study reconfirmed findings from previous studies and showed that Ang II does induce an elevation in TLR4 expression in the heart.

As a key cell type in the heart, cardiomyocytes serve as a potential target for inflammation. TLR2, TLR3, and TLR4 are the TLRs that are predominately expressed in cardiomyocytes and play a large role in mediating inflammatory responses that are associated with cardiovascular disease (21). However, there are no known studies providing evidence for the role of TLR4 inhibition in cardiomyocytes. To determine the cardiomyocyte specific role of TLR4, we generated a cardiomyocyte specific TLR4 knockout mouse. After confirmation of this genetic deletion, we implanted mice with Ang II for 14 days. Blood pressure recordings showed that cTLR4KO mice have an attenuated mean arterial pressure when treated with Ang II compared with their WT counterparts. However, the blood pressure is still significantly elevated in cTLR4KO mice after Ang II-infusion suggesting that there are other mechanisms or factors are involved in this response. Further studies are necessary to understand the complex interplay of hypertensive mechanisms.

As a result of hypertension, the heart must physiologically adapt to the increased demand, and will stimulate myocyte hypertrophy, collagen formation, and remodel the myocardium with a disproportionate increase in fibrous tissue (27). Thus, we measured cardiac hypertrophy related to Ang II hypertension by measuring heart weight to body weight ratio. Ang II for 14 days not only increased blood pressure, but also led to cardiac hypertrophy indicated by increased heart weight to body weight ratios. In contrast, cTLR4KO mice were protected from this cardiac hypertrophy and showed a reduced response to Ang II. To specifically determine the changes in cardiomyocytes during this hypertrophic remodeling, we examined cardiomyocyte hypertrophy and found that cTLR4KO mice, unlike WT mice, had an attenuation of Ang II-induced cardiomyocyte specific hypertrophy. Additionally, we measured hypertrophic markers ANP, BNP and B-MHC. Once again, infusion of Ang II produced an increase in gene expression of all three markers, and cTLR4KO mice showed an attenuated response. These findings suggest that cTLR4KO mice are protected from Ang II-induced cardiac hypertrophy.

Cardiac fibrosis is characterized by an increase in extracellular matrix (ECM) components and collagens in heart muscle and is a crucial component of pathological hypertrophy (33). To observe whether cTLR4KO mice are protected from cardiac fibrosis we measured the gene expression of profibrotic collagen I, III and connective tissue growth factor. We found that Ang II increased the expression of these profibrotic markers in WT mice, but this increase was mitigated in cTLR4KO mice. To then determine fibrosis levels as evidenced by collagen deposition, we stained heart sections from these mice with masson's trichome staining and showed that Ang II increased collagen deposition. Again, cTLR4KO mice showed a decrease in collagen staining in the left ventricle, suggesting that cTLR4KO mice have an attenuation of fibrosis.

Cardiac fibrosis may result in deviations in matrix composition and standards while also leading to impaired heart muscle function. Using echocardiography, we measured parameters of cardiac function and observed a deterioration of LV function in the Ang II treated WT mice as indicated by increased IVSd, LVPWTd and decreased fractional shortening. In cTL4KO mice, there was an attenuation of these effects, indicating that cTLR4KO mice had a preserved cardiac function when compared with WT mice. The attenuation of the deleterious effects on cardiac function within the cTLR4KO mice provides additional evidence that cTLR4KO reduces the burden of hypertension on the heart.

Recent studies have reported that cardiac inflammation induced by Ang II may occur through the TLR4/MyD88/NF-κB and TLR4/TRIF/NF-κB pathways (14, 19, 32, 39). NF-κB is a known transcription factor involved in TLR signaling pathways. In cardiomyocytes, Ang II-induced TLR4 activation of NF-κB in a MyD88dependent manner increases release of proinflammatory cytokines and further results in inflammation and hypertension (5, 43). In Ang II-induced cardiac hypertrophy, NF-κB is activated by the TRIF adaptor protein which mediates responses to both TLR3 and TLR4 (34). To confirm this in our study, we measured NF-κB levels and showed that Ang II increases NF-κB activity in WT mice, and this effect was attenuated in cTLR4KO mice, allowing us to believe that Ang II-induced hypertension occurs through the MyD88 dependent and independent TLR4/NF-κB signaling pathways in cardiomyocytes.

Although a rise in inflammation is critical in times of infection and injury, chronic inflammation has been implicated in the etiology of various diseases such as cancer, diabetes mellitus, neurodegenerative disorders, and cardiovascular disease (13). Previously, we showed the anti-inflammatory effect of TNF in the brain in Ang II-induced hypertension (35, 36). Additionally, numerous studies have demonstrated that inflammation is a pivotal component of Ang II-induced hypertension, especially within the heart (23). To determine whether cTLR4KO mice are protected from inflammation, we measured proinflammatory cytokine gene expression for TNF, IL-1β, IL-6 and MCP-1. We found that cTLR4KO mice are protected from Ang II-induced proinflammatory cytokine upregulation when compared with WT mice. Additionally, serum ELISA of TNF and IL-1β provide sufficient supporting evidence that cTLR4KO mice have an attenuation in these proinflammatory cytokines resulting in protection from inflammation.

As an initial step to study the contribution of the TLR4 pathway in the development of Ang II-induced hypertension and cardiac remodeling, the present study was conducted only in male mice. Further studies in female mice are warranted to determine whether sex differences exist regarding the expression of TLR4 in cardiomyocytes of hypertensive hearts and specific contribution of cardiomyocyte TLR4 inhibition on hypertension and cardiovascular function. To conclude, our study reveals an important role for cardiomyocyte specific deletion of TLR4 in protection from Ang II-induced hypertension by reducing cardiac hypertrophy, fibrosis, and blunting inflammation. This study further defines the role of cardiomyocyte TLR4 inhibition as a prospective target for future antihypertensive therapeutics.

## Data Availability

The original contributions presented in the study are included in the article/Supplementary Material, further inquiries can be directed to the corresponding author/s.
